# Distinct Modulation of Spontaneous and GABA-Evoked Gating by Flurazepam Shapes Cross-Talk Between Agonist-Free and Liganded GABA_A_ Receptor Activity

**DOI:** 10.3389/fncel.2018.00237

**Published:** 2018-08-28

**Authors:** Magdalena Jatczak-Śliwa, Katarzyna Terejko, Marek Brodzki, Michał A. Michałowski, Marta M. Czyzewska, Joanna M. Nowicka, Anna Andrzejczak, Rakenduvadhana Srinivasan, Jerzy W. Mozrzymas

**Affiliations:** ^1^Laboratory of Neuroscience, Department of Biophysics, Wrocław Medical University, Wrocław, Poland; ^2^Department of Molecular Physiology and Neurobiology, University of Wrocław, Wrocław, Poland

**Keywords:** GABA_A_ receptor, γ-aminobutyric acid, benzodiazepines, spontaneous activity, preactivation, gating, partial agonist

## Abstract

GABA_A_ receptors (GABA_A_Rs) play a crucial inhibitory role in the CNS. Benzodiazepines (BDZs) are positive modulators of specific subtypes of GABA_A_Rs, but the underlying mechanism remains obscure. Early studies demonstrated the major impact of BDZs on binding and more recent investigations indicated gating, but it is unclear which transitions are affected. Moreover, the upregulation of GABA_A_R spontaneous activity by BDZs indicates their impact on receptor gating but the underlying mechanisms remain unknown. Herein, we investigated the effect of a BDZ (flurazepam) on the spontaneous and GABA-induced activity for wild-type (WT, α_1_β_2_γ_2_) and mutated (at the orthosteric binding site α_1_F64) GABA_A_Rs. Surprisingly, in spite of the localization at the binding site, these mutations increased the spontaneous activity. Flurazepam (FLU) upregulated this activity for mutants and WT receptors to a similar extent by affecting opening/closing transitions. Spontaneous activity affected GABA-evoked currents and is manifested as an overshoot after agonist removal that depended on the modulation by BDZs. We explain the mechanism of this phenomenon as a cross-desensitization of ligand-activated and spontaneously active receptors. Moreover, due to spontaneous activity, FLU-pretreatment and co-application (agonist + FLU) protocols yielded distinct results. We provide also the first evidence that GABA_A_R may enter the desensitized state in the absence of GABA in a FLU-dependent manner. Based on our data and model simulations, we propose that FLU affects agonist-induced gating by modifying primarily preactivation and desensitization. We conclude that the mechanisms of modulation of spontaneous and ligand-activated GABA_A_R activity concerns gating but distinct transitions are affected in spontaneous and agonist-evoked activity.

## Introduction

GABA(gamma aminobutyric acid) is the major inhibitory neurotransmitter in the brain (Cherubini and Conti, 2001; Fritschy and Brünig, 2003). BDZs are positive modulators of specific subtypes of GABA_A_Rs (Rudolph and Möhler, 2004), but the mechanism of action of BDZs remains obscure and the controversy is whether BDZs act primarily on the agonist binding step or on gating or on both. In our early studies, we proposed that BDZ receptor agonists affected binding and desensitization (Mozrzymas et al., 2007). Li et al. (2013) reported that BDZs altered the efficacy, and similar conclusions were proposed by Rusch and Forman (2005) and Campo-Soria et al. (2006). More recently, Dixon et al. (2015) proposed that BDZs affected the intermediate conformation, whereas Goldschen-Ohm et al. (2014) concluded that BDZs affected the binding and preactivation (flipping) steps. Thus, although the impact of BDZs on gating transitions is emerging, we decided to investigate which specific transitions are modulated.

Positive modulation of spontaneous activity of WT receptors by BDZs (Campo-Soria et al., 2006) and of mutants favoring spontaneous activity (Downing et al., 2005; Rusch and Forman, 2005; Campo-Soria et al., 2006) indicated an interference with gating. However, the mechanism of the impact of BDZs on spontaneous activity remains unknown. In particular, it is not clear whether BDZs directly activate GABA_A_Rs or upregulate the spontaneous openings (Downing et al., 2005; Campo-Soria et al., 2006). Moreover, in aforementioned studies, mutations enhancing spontaneous activity were located close to the channel gate, affecting the receptor gating probably at its latest stage, whereas the impact of BDZs could occur earlier. It is possible that mutations located at different sites could result in different molecular scenarios of spontaneous activity. It thus seems interesting to investigate the mutations that are likely to affect the early stages of GABA-induced activation but still result in enhanced spontaneous activity. Considering these premises, we decided to provide a comprehensive description of the effect of BDZ on the spontaneous activity and its impact on GABA-induced currents.

Herein, we address the mechanism of FLU action on GABA_A_Rs by considering α_1_F64 mutants in which gating (primarily preactivation) is affected and by using full and partial (P4S) agonists activating WT receptors. Surprisingly, in spite of their localization at the binding site, these mutations strongly increase spontaneous activity. FLU upregulates the spontaneous activity of both WT receptors and α_1_F64 mutants by affecting opening/closing transitions. We provide a mechanistic description of how FLU affects the cross-talk between spontaneous and GABA-induced activity. Based on our data and model simulations, we propose that FLU affects agonist-induced gating by modifying primarily preactivation and desensitization.

## Materials and Methods

### Transfection and Expression of Recombinant GABA_A_Rs

The experiments were performed on human embryonic kidney (HEK-293) cells cultured as described in Szczot et al. (2014). Twenty-four hours prior to transfection, the cells were replated on poly-d-lysine (1 μg/ml) coated coverslips (Carl Roth, Karlsruhe, Germany). A standard calcium phosphate precipitation method (Chen and Okayama, 1987) was used to transiently transfect the cells. When stronger expression was needed, FuGENE HD (Promega, Madison, WI, United States) at a 3:1 FuGENE HD:DNA ratio was used. cDNA encoding rat GABA_A_R subunits was cloned in a pCMV vector. The α_1_/β_2_/γ_2L_ subunits were mixed in the ratio of 1:1:3 (0.5:0.5:1.5 μg) in the transfected solution, together with 0.5 μg human cluster of differentiation 4 (CD4) encoding plasmid. This amount of cDNA was used per four coverslips. Recordings in the whole-cell or out-side out configurations were performed 24–48 h after transfection. To successfully detect the transfected cells, Dynabeads CD4 magnetic binding beads were used (Invitrogen, Carlsbad, CA, United States).

### Patch Clamp Recordings, Perfusion Systems, and Macroscopic Data Analysis

Currents were low-pass filtered at 10 kHz and recorded at a holding potential of -40 mV using an Axopatch 200B amplifier (Molecular Devices, Sunnyvale, CA, United States) and acquired using a Digidata 1550A acquisition card (Molecular Devices, Sunnyvale, CA, United States). For signal acquisition, pClamp 10.7 software (Molecular Devices, Sunnyvale, CA, United States) was used. Pipettes were pulled from borosilicate glass (OD: 1.5 mm, ID: 1.0 mm; Hilgenberg, Malsfeld, Germany) and filled with an intracellular solution containing (in mM) 137 KCl, 1 CaCl_2_, 2 ATP-Mg, 2 MgCl_2_, 10 K-gluconate, 11 EGTA, and 10 HEPES (pH adjusted to 7.2 with KOH). The pipette resistance ranged between 3 and 6 MΩ. Standard Ringer’s solution was used as an external saline containing (in mM) 137 NaCl, 5 KCl, 2 CaCl_2_, 1 MgCl_2_, 10 HEPES, and 20 glucose (pH to 7.2 with NaOH). To avoid osmolarity imbalance, for agonist concentrations >10 mM, adjustments in reagents concentration were made as described previously by Szczot et al. (2014).

An ultrafast perfusion system using theta-glass capillaries (Hilgenberg, Malsfeld, Germany) mounted on a piezoelectric-driven translator (Physik Instrumente, Karlsruhe, Germany) was used as described in detail by Jonas (1995) and by our group (Mozrzymas et al., 2003, 2007; Szczot et al., 2014). Solutions were supplied simultaneously to the two channels of the theta-glass capillary with a high-precision SP220IZ syringe pump (World Precision Instruments, Inc., Sarasota, FL, United States). The solution exchange time, measured with the open-tip capillary, ranged from 100 to 350 μs, depending on the size of the theta glass and the flux speed. When such a rapid exchange was not necessary and when more elaborate protocols were needed (requiring a larger number of channels), a multibarrel rapid solution changer RSC-200 (Bio-Logic Science Instruments, Seyssinet-Pariset, France) was used. Recording on adherent cells showed the highest stability and this recording mode was used for protocols requiring several applications of different solutions (exchange time approximately 20–30 ms), but it was limited to slow signals.

The FLU effect was assessed in terms of the relative values determined in the presence of this drug and in control conditions at saturating [GABA]. The saturating concentration of GABA for WT and mutated α_1_β_2_γ_2_ GABA_A_R (at α_1_F64 residue) was previously determined by our group (Szczot et al., 2014) and was established as 100 mM [GABA] for leucine (LEU) and alanine (ALA) mutants, but this concentration was not fully saturating for the cysteine (CYS) mutant (Kisiel et al., 2018). In all experiments testing the impact of FLU, this compound was used at a concentration of 3 μM. For the majority of recordings, a pretreatment protocol was used in which FLU was present both in the wash solution (Ringer’s solution) and the agonist-containing solution. In a part of the experiments, a co-application protocol was used, where FLU was present in the agonist-containing solution but absent in the wash. The two protocols were used to investigate the possible differences in the effect of FLU pretreatment on an agonist-evoked receptor activation (see section “Results”). To study the receptor deactivation time course, two experimental protocols were used: a short pulse of saturating [GABA] whose duration was sufficient to reach the amplitude peak (determined for each mutant separately) and a long (500 ms) agonist application.

The current onset was measured as 10–90% rise time (RT). The kinetics of deactivation was analyzed on normalized traces (amplitude of deactivating current equal to 1) and described in terms of the time constant(s) obtained from either a single exponent: y(t) = A⋅exp(-t/τ) where A is a unitary amplitude and τ is the time constant, or from a sum of two exponential functions: y(t) = A_slow_⋅exp(t/τ_slow_) + A_fast_⋅exp(t/τ_fast_) where A_slow_ and A_fast_ are the amplitude percentages of slow and fast components, respectively (A_slow_ + A_fast_ = 1), and τ_slow_ and τ_fast_ are the time constants. In the case of two components, the mean time constant (τ_mean_) was calculated as τ_mean_ = A_slow_⋅ τ_slow_ + A_fast_⋅ τ_fast_.

Macroscopic desensitization kinetics was described using exponential fitting (typically two components, denoted as τ_fast_ and τ_slow_) with a constant value. In the case of currents, for which exponential fitting was problematic (due to, e.g., slow changes), the desensitization onset was quantified as a total amplitude fraction remaining after 10 ms (abbreviated FR10). The extent of desensitization was quantified as a total amplitude fraction remaining after 500 ms (abbreviated FR500).

**FIGURE 1:**
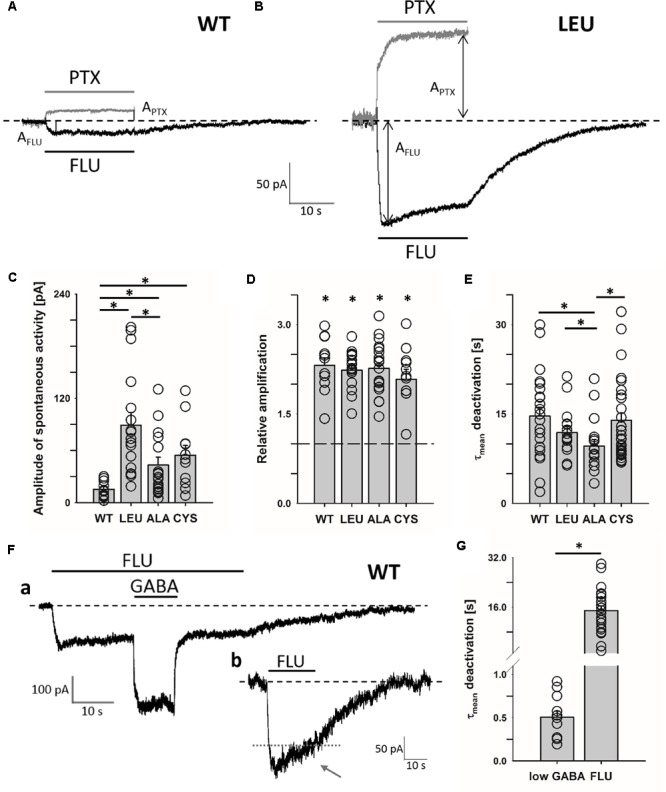
α_1_F64 mutations increase spontaneous activity of GABA_A_R and FLU potentiates this activity to the same extent as for WT receptors. Typical traces for WT receptor **(A)** and LEU mutant. **(B)** mediated current responses for 3 μM FLU (black) and 100 μM PTX (gray) applications. Amplitudes are marked with arrows and the baseline level with a dashed line. **(C)** Statistics for the amplitudes of spontaneous activity for WT and mutated receptors (WT: 15.5 ± 2.8 pA, *n* = 13; LEU: 89.0 ± 14.5 pA, *n* = 17; ALA: 43.3 ± 9.6 pA, *n* = 22; CYS: 54.6 ± 11.5 pA, *n* = 11). **(D)** Relative enhancement of spontaneous activity by FLU determined as (A_FLU_ + A_PTX_)/A_PTX_, where A_FLU_ is the amplitude of FLU-evoked current and A_PTX_ is the amplitude of spontaneous activity determined by PTX application (see section “Materials and Methods”) (WT: 2.32 ± 0.13, *n* = 12; LEU: 2.24 ± 0.08, *n* = 17; ALA: 2.27 ± 0.09, *n* = 21; CYS: 2.08 ± 0.17, *n* = 11). **(E)** Comparison of τ_mean_ for deactivation after FLU application for WT and mutants (WT: 14.7 ± 1.5 s, *n* = 23; LEU: 11.9 ± 0.99 s, *n* = 16; ALA: 9.6 ± 1.1 s, *n* = 17; CYS: 14 ± 1.2 s, *n* = 35). **(F)** Typical traces of WT GABA_A_R-mediated current responses: **(a)** for FLU and 100 nM of GABA (note the large difference in deactivation kinetics after low [GABA] and FLU removal), **(b)** exemplary trace illustrating slow desensitization onset seen as a current fading (gray dotted line added to reveal the extent of fading during FLU application). **(G)** Statistics for WT τ_mean_ deactivation after low [GABA] (0.51 ± 0.07 s, *n* = 24) and FLU (14.7 ± 1.5 s, *n* = 12) removal. All recordings were performed using the Bio-Logic system in the whole-cell configuration. Asterisks show a statistically significant difference.

For the CYS mutant, which exhibited the slowest kinetics, a part of results (current amplitude measurements) was obtained using a Bio-Logic perfusion system using analogous protocols as in the theta-glass experiments. The results of the experiments carried out using these two systems were consistent, and the data were pooled. Experiments in which the extent of spontaneous activity was assessed by applying the open channel blocker picrotoxin (PTX) at a concentration of 100 μM were carried out using the Bio-Logic perfusion system. The extent of enhancement of the spontaneous activity by FLU was assessed using the following protocol: first FLU was applied and, after washout, PTX was administered and the extent of amplification was calculated as (A_PTX_ + A_FLU_)/A_PTX_, where A_PTX_ is the amplitude shift after PTX application and A_FLU_ is the amplitude of current appearing upon FLU application (with respect to the baseline in the absence of PTX, **Figures 1A,B**).

All electrophysiological recordings were conducted at room temperature (20–23°C). All chemicals were purchased from Sigma-Aldrich (St. Louis, MO, United States) unless stated otherwise.

### Single-Channel Recordings

Single-channel currents were recorded using the patch-clamp technique in the cell-attached mode. Signals were amplified by an Axopatch 200B amplifier (Molecular Devices, Sunnyvale, CA, United States) and digitized by a Digidata 1550B acquisition system (Molecular Devices, Sunnyvale, CA, United States) with a 100 kHz sampling rate. Signals were initially filtered at 10 kHz with a low-pass Bessel filter built-in in the amplifier. The pipette potential was set at 100 mV. Patch pipettes with tip resistance of 6–12 MΩ were pulled from thick-walled, filamented borosilicate glass (OD: 1.5 mm, ID: 0.87 mm; Hilgenberg, Malsfeld, Germany) on a P-1000 horizontal puller (Sutter Instrument, Novato, CA, United States). Pipettes were coated with Sylgard 184 (Dow Corning, Auburn, MI, United States) and fire-polished on a microforge. To minimize the signal noise, the amount of the extracellular solution in the dish (35 mm Ø Nunclon, Thermo Fisher, Waltham, MA, United States) was kept at 1 ml, yielding minimal immersion of the recording electrode. For the same reason, only patches with resistance exceeding 10 GΩ were considered suitable for experiments. The extracellular and intrapipette solution consisted of (in mM) 102.7 NaCl, 20 Na-gluconate, 2 KCl, 2 CaCl_2_, 1.2 MgCl_2_, 10 HEPES (Roth), 20 TEA-Cl, 14 D-(+)-glucose, and 15 sucrose (Roth), prepared in deionized water. The pH was adjusted to 7.4 with 2M NaOH. Prior to single-channel recordings, control experiments were performed on HEK cells not transfected and those transfected with only CD4 plasmids. In these recordings, we observed very rare, mostly long-lasting openings, but the channels mediating these events had a much lower conductance than α_1_β_2_γ_2_ GABA_A_ receptors and thereby their interference could be easily eliminated from our analysis.

### Analysis of Single-Channel Currents

Recorded signals were additionally filtered off-line (8-pole low-pass Bessel filter) using Clampex 10.7 software (Molecular Devices, Sunnyvale, CA, United States) to achieve a signal-to-noise ratio of at least 15:1. Subsequently, the sampling rate was reduced to maintain a 10:1 sampling-to-filter frequency ratio. To reduce the probability of analyzing the recordings from a multitude of channels, recordings with a high activity and/or ones with visible multiple openings were excluded from further analysis. The idealization of single-channel activity was performed with the SCAN software (DCProgs^1^) and idealization files were processed in EKDIST (DCProgs^1^) to create dwell-time distributions, for open and shut events that were then fitted with the sums of exponentials and the respective time constants (τ) and percentages (P) were determined. The DCProgs software package has been kindly given to our group by David Colquhoun (UCL London).

### Data and Statistical Analysis

All macroscopic current recordings were analyzed using pClamp 10.7 software (Molecular Devices, Sunnyvale, CA, United States). Only the recordings that did not exceed 20% of signal instability (most often rundown) were qualified for the statistical analysis, which was performed using Excel 2016 (Microsoft, Redmond, WA, United States) and SigmaPlot 11.0 (Systat Software, San Jose, CA, United States). Data comparison was performed using the Student’s *t*-test preceded by the Shapiro–Wilk normality test or the Wilcoxon signed-rank test and the Mann–Whitney *U*-test for the data that failed normality assessment. The statistical significance threshold was defined as *p* < 0.05.

### Homology Modeling and Ligand Docking

The structural homology model of α_1_β_2_γ_2_ GABA_A_R was constructed using a similar approach as that described by Michałowski et al. (2017), but a distinct structural template was selected: a homomeric β_3_ GABA_A_R crystal structure (Miller and Aricescu, 2014) instead of a glycine receptor (Du et al., 2015). Prior to model construction, the sequence alignment of α_1_, β_2_, γ_2_, and β_3_ GABA_A_R subunits and sequences of other pentameric ligand gated ion channels (pLGICs) was performed in T-Coffee (Notredame et al., 2000) and refined manually using Jalview (Waterhouse et al., 2009). The MODELLER Python package (Šali and Blundell, 1993) was used to construct 1,000 initial α_1_β_2_γ_2_ GABA_A_R structures. Among these, the best ones were selected according to the MODELLER quality estimates DOPE and molpdf and evaluated using RAMPAGE (Lovell et al., 2003). To further improve their quality, an iterative protocol of modeling using the MODELLER’s refinement function “loopmodel” was employed. Briefly, the major part of the receptor model was constrained and areas of positions from the Ramachandran outlier region were remodeled until satisfactory quality was achieved. The final model was assessed with the Ramachandran plot evaluation, showing no residues in the outlier region (97.8 and 2.2% in favored and allowed regions, respectively). The ligands investigated in the present study were docked to the obtained homology model. The structures of GABA, P4S, and FLU were taken from the Zinc database (Irwin et al., 2012) and initially inserted into their respective binding sites according to the binding position of benzamidine in the experimental homomeric β_3_ GABA_A_R crystal structure (Miller and Aricescu, 2014). The final binding positions and the binding energy were obtained by the AutoDock Vina (Trott and Olson, 2010) flexible fit docking method. Each ligand was docked several times and the best binding position was selected according to the energy level and the properties of amino acids building the binding site. Upon docking, the free energy of binding (ΔG, [kcal/mol]) was estimated for each ligand. Analysis of the results and visualizations were performed using VMD (Humphrey et al., 1996) and Python scripts.

### Kinetic Scheme Modeling

Spontaneous activity was described using the kinetic scheme proposed by Kisiel et al. (2018), but to describe the modulation by FLU, an additional binding step for this modulator was added and the remaining rate constants were reassessed. To estimate the FLU binding/unbinding rates, ligand docking was used and the dissociation constant (K_D_) was calculated using **Eq. 1** (ΔG: binding energy, R: gas constant, T: temperature) as follows.

(1) $$\begin{array}{c} K_{D}{=e}^{\frac{\Delta G}{\mathrm{RT}}} \end{array}$$

(2) $$\begin{array}{c} K_{D}=\frac{k_{\mathrm{off}}}{k_{\mathrm{on}}} \end{array}$$

Having established the ratio of the unbinding and binding rates (**Eq. 2**, k_on/off_: binding/unbinding rates), the absolute values of these rate constants were optimized to reproduce the experimentally observed RT and deactivation kinetics upon FLU application (**Figure 9A**). The remaining rates were estimated according to single-channel open time distributions. In addition, to reproduce the kinetics of responses to exogenous FLU applications and overshoot, the selected rates were altered, but the resultant mean open times remained preserved. To describe GABA and P4S-evoked activity of the mutated receptor and its modulation by FLU, the kinetic scheme of the flipped Jones–Westbrook model from Szczot et al. (2014) was employed. It needs to be underlined that this model was optimized to fit the current responses recorded in the excised patch outside-out configuration, whereas in the present study we have collected data in the whole-cell mode because these recordings were more stable and had larger amplitudes and this was advantageous especially when recording currents mediated by mutants and/or evoked by the partial agonist. However, due to the slower solution exchange in the whole-cell configuration, the current onset, which was the fastest response characteristic, was slower than that measured in the outside-out patches. Nevertheless, all the effects of FLU on current responses (including the acceleration of current onset) observed on the excised patches were well-reproduced in the whole-cell recordings. In this situation, formal fits to the rising phase of current traces measured in the whole-cell configuration would lead to the misinterpretation of the slower current onset (than in excised patches) as slower binding and/or gating features underlying onset kinetics. Considering these premises, we employed the binding rate constants estimated in our previous study (Szczot et al., 2014) as control conditions and modified the rate constants to reproduce the relative impact of FLU on GABA_A_Rs. To reproduce both fast and slow components of macroscopic desensitization, it was necessary to incorporate an additional slow desensitizing state (**Figure 8C**, A_2_D′ state). To describe the changes in kinetics of mutated receptors and in their modulation by FLU, the rate constants were adjusted to best reproduce the trends observed experimentally (mainly: amplitude, RT change trend, FR10, FR500, and/or desensitization and deactivation time constants). Adding the second desensitized state led to the construction of a more extensive model in which we had to consider a possibility that multiple sets of transition rates would result in similar simulated traces. Thus, to reduce the degrees of freedom, according to preliminary simulations and data from literature, some of the rates were fixed. The β and α rates were fixed, because these rates were not affected by CYS and LEU mutations (Szczot et al., 2014) at saturating [GABA]. Moreover, WT receptor modulation by FLU at saturation is relatively weak; hence, we did not expect any significant impact of FLU on A_2_O-associated rates. On the other hand, the preactivation rates (δ, γ), highly influenced by mutations, were varied to obtain the optimal fit. In addition, in all receptors, FLU changed the amplitude and accelerated the onset kinetics. This effect could be only reproduced by the changes in flipping rates, but not A_2_O-associated rates. Iterative simulations were performed using custom Python scripts and ChanneLab 2.0 (Synaptosoft, Decatur, GA, United States) and the parameters describing the simulated current time course were calculated in pClamp 10.7 software (Molecular Devices, Sunnyvale, CA, United States).

## Results

### Flurazepam Affects Spontaneous Activity of WT Receptors and α_1_F64 Mutants

It has been reported that various types of GABA_A_Rs, including α_1_β_2_γ_2_, show spontaneous activity that can be enhanced by BDZs (Mortensen et al., 2003), but the underlying mechanism remains unknown. Interestingly, for GABA-evoked currents, Gielen et al. (2012) and Dixon et al. (2015) suggested the impact of BDZs on the preactivation, but our recent report indicated that spontaneous openings do not require the preactivation step (Kisiel et al., 2018). To explore this problem, we compared the BDZ sensitivity of spontaneous activity mediated by WT receptors and by α_1_F64 mutants in which the preactivation step is impaired (Szczot et al., 2014; Kisiel et al., 2018). As expected, the WT receptor showed a low spontaneous activity revealed by 100 μM PTX (**Figures 1A,C**), but the mutations at the α_1_F64 residue strongly increased it (**Figures 1B,C**). There was no statistically significant differences in the extent of potentiation by FLU on WT receptors and all considered mutants (LEU, ALA, CYS, **Figure 1D**). The onset of currents observed after FLU application and mediated by WT receptors was very slow (1.63 ± 0.15 s, *n* = 22) and for the α_1_F64 mutants it was slightly (but significantly) faster (1.09 ± 0.07 s, *n* = 16 for LEU; 1.12 ± 0.08 s, *n* = 17 for ALA; and 1.26 ± 0.01 s, *n* = 35 for CYS, data not shown). These currents had an extremely slow deactivation after FLU removal (**Figures 1A,B,E**) that we found to be correlated with FLU binding energetics (see section “Model Simulations”). When studying the spontaneous activity, a precaution should be taken to rule out trace contaminations by GABA. We have thus additionally tested applications of low GABA concentration (1–100 nM, with and without FLU present in the bath). As shown in **Figures 1Fa,G**, currents following FLU application and responses to 100 nM GABA had deactivation kinetics differing by nearly two orders of magnitude (**Figure 1G**, similar proportions were observed when GABA was applied alone, data not shown). Thus, if there was any trace contamination from GABA (wash, FLU- and GABA-containing solutions were prepared with the same Ringer’s saline), then a rapid component would be expected also after FLU removal, but that was not observed. It is worth noting that in the case of prolonged exposure to FLU, an initial current onset is followed by a weak but clear fading, indicating the desensitization of the unbound receptor (**Figure 1Fb**, arrow).

### Flurazepam Affects Open Times of Spontaneous Openings

**FIGURE 2:**
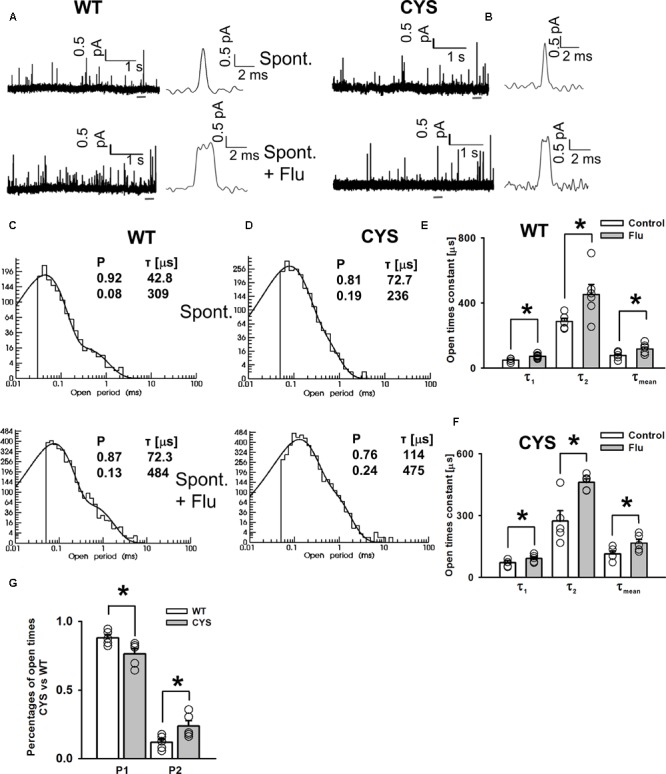
FLU enhances spontaneous GABA_A_R activity by prolonging single-channel openings. Typical spontaneous currents mediated by WT **(A)** and CYS mutant **(B)** GABA_A_R (upper traces) and openings in the presence of FLU (lower traces), presented in different time scales. **(C,D)** Typical open time distributions for WT receptor **(C)** and CYS mutant. **(D)** Spontaneous events (upper) and events in the presence of FLU (lower). Insets show the fitting parameters for these distributions. **(E)** Open time constants for spontaneous activity of WT receptors (white bars, *n* = 6) and in the presence of FLU (gray bars, *n* = 5). **(F)** Open time constants for spontaneous activity of CYS mutants (white bars, *n* = 5) and in the presence of FLU (gray bars, *n* = 5). **(G)** Comparison of component percentages of open time distributions for events mediated by WT receptors and CYS mutants. For WT receptors and CYS mutants, percentages of open time components were not affected by FLU (not displayed). All recordings were performed in the cell-attached configuration. Asterisks show a statistically significant difference.

To further explore the modulation of spontaneous events by FLU, we conducted single-channel recordings as described by Kisiel et al. (2018; see section “Materials and Methods”). Considering the similarity in the FLU effect on WT receptors and mutants, we limited these recordings to the WT and the CYS mutant (**Figures 2A,B**), which is most distinct from the native receptor (Kisiel et al., 2018). Open time distributions for WT and CYS receptors had two components with time constants that did not differ between these receptor types, but for CYS mutants the percentage of the slower component was larger (**Figures 2C,D,G**). Interestingly, FLU produced a similar effect on the activity of the WT and CYS mutants by significantly increasing both time constants (**Figures 2C–F**) and thereby the mean open time (**Figures 2E,F**). The analysis of closed times did not reveal any major difference but it was problematic as spontaneous activity did not show bursts (formally the number of openings per burst was invariably 1) Kisiel et al., 2018), excluding any assessment of the number of channels in the patch.

### Cross-Talk of Spontaneous and GABA-Evoked Activity

**FIGURE 3:**
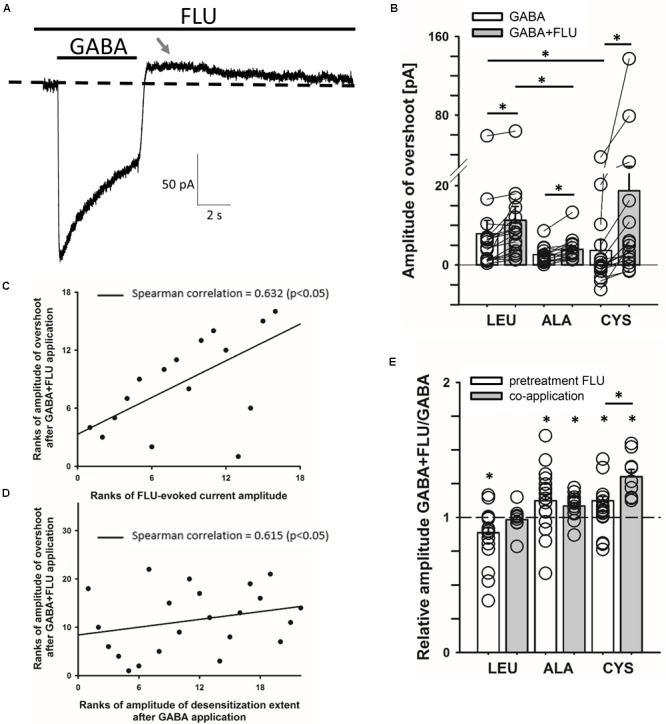
Cross-talk between spontaneous and GABA-induced activity gives rise to the overshoot. **(A)** Typical trace of the current response mediated by the CYS mutant showing overshoot (marked with gray arrow) after saturating [GABA] (100 mM) application with FLU present in the bath (pretreatment protocol). Baseline level is marked with dashed line. **(B)** Statistics for overshoot amplitudes for mutated receptors after GABA or GABA+FLU application in the pretreatment protocol (for GABA: LEU: 7.9 ± 3.3 pA, *n* = 17, ALA: 2.6 ± 0.6 pA, *n* = 13, CYS: 3.6 ± 2.7 pA, *n* = 16; for GABA+FLU: LEU: 11.3 ± 3.5 pA, *n* = 17, ALA: 3.9 ± 0.9 pA, *n* = 13, CYS: 18.7 ± 9.4 pA, *n* = 16). Overshoot was positive when current crossed the baseline and negative when the current did not reach baseline. **(C,D)** Plots showing significant Spearman correlation between overshoot amplitudes after GABA+FLU application and **(C)** amplitudes of currents observed upon FLU application (**Figure 1**), **(D)** amplitudes of desensitization extent after GABA application (the remaining current amplitude form the peak to the end of agonist application). **(E)** Comparison of the relative FLU effect on the amplitude of current responses evoked by saturating [GABA] in pretreatment (white bars) and co-application protocol (gray bars) (for pretreatment: LEU: 0.87 ± 0.04, *n* = 29, ALA: 1.12 ± 0.05, *n* = 18, CYS: 1.12 ± 0.03, *n* = 32; for co-application: LEU: 0.98 ± 0.03, *n* = 9, ALA: 1.09 ± 0.02, *n* = 16, CYS: 1.3 ± 0.05, *n* = 9). All recordings were performed in the whole-cell configuration using the ultrafast perfusion system (theta glass) for LEU and ALA or the Bio-Logic system for CYS. Asterisks show a statistically significant difference.

We asked whether the modulation of spontaneous activity by FLU might affect the GABA-elicited activity. When BDZs are tested, cells are typically pretreated with these drugs prior to the application of agonist and the “baseline” is commonly interpreted as the “no activity” reference which, because of spontaneous activity, is problematic. In **Figure 3A**, a typical response to saturating [GABA], in the presence of FLU, mediated by the CYS mutants (pretreatment protocol) is shown. It is evident that after GABA removal, deactivation is followed by an overshoot (arrow in **Figure 3A**). Although in the case of WT receptors, the spontaneous activity was weak (**Figure 1C**), in cells with strong expression, the overshoot could be also seen (data not shown), indicating a common mechanism. As we discuss it in Section “Model Simulations,” this phenomenon reflects a cross-desensitization of spontaneous and ligand-activated receptors. The following evidence indicates the involvement of spontaneous activity and its modulation by FLU in the overshoot phenomenon. The enhancement of spontaneous activity by FLU is associated with an increased overshoot (**Figure 3B**). This relationship is further supported by a significant correlation between the amplitudes of the overshoot and the amplitudes of currents observed upon the application of FLU (**Figure 3C**). Moreover, in the case of the CYS mutant, we observed a clear correlation between GABA-induced macroscopic desensitization and the amplitude of the overshoot (**Figure 3D**). Considering thus a potential impact of spontaneous activity on GABA-evoked responses, we compared the amplitudes of currents evoked by saturating [GABA] using the pretreatment and co-application protocols. As is shown in **Figure 3E**, the relative current amplitude measured using the co-application protocol was different from that upon FLU pretreatment.

### Impact of FLU on GABA-Evoked Responses Mediated by α_1_F64 Mutants

**FIGURE 4:**
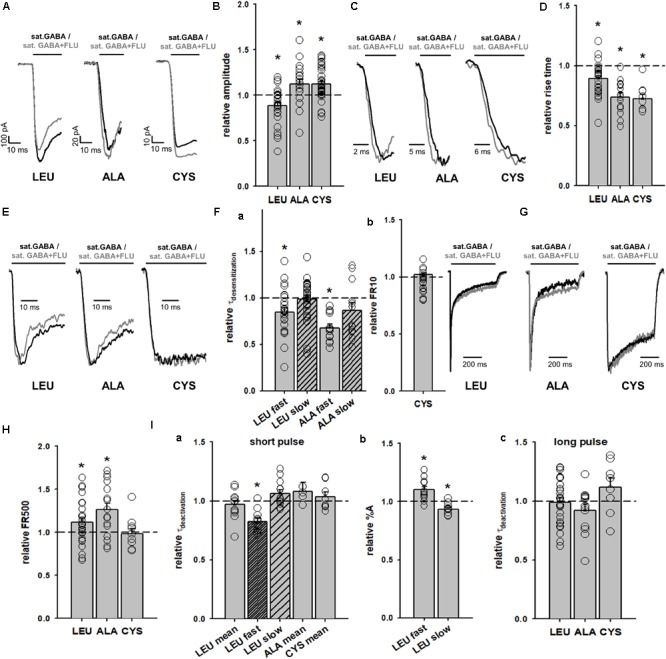
FLU affects both amplitudes and kinetics of currents mediated by α_1_F64 mutants. **(A)** Typical traces of current responses to saturating [GABA] application (black traces) and to saturating GABA+FLU (gray traces, pretreatment protocol) for LEU, ALA, and CYS mutants. **(B)** Statistics for the relative FLU effect on current amplitude (for relative values see **Figure 3**). **(C)** Typical normalized traces of the rising phases of currents evoked by saturating [GABA] in control conditions (black line) and in the presence of FLU (gray lines). **(D)** Statistics for the relative FLU effect on the RT. Absolute control RT values (for GABA: LEU: 3.08 ± 0.15 ms, *n* = 26; ALA: 6.10 ± 0.55 ms, *n* = 14; CYS: 9.07 ± 1.22 ms, *n* = 9). **(E)** Typical normalized traces revealing the onset of macroscopic desensitization for mutants, observed upon prolonged application of GABA (black traces) and in the presence of FLU (gray traces). **(F)** Statistics for desensitization kinetics: **(a)** relative values of the time constants in the presence of FLU for LEU and ALA mutants [absolute control values for GABA: LEU: 7.92 ± 0.81 (τ_fast_), A_fast_: 0.38 ± 0.02, *n* = 27; 170; 99 ± 0.04 ms (τ_slow_), A_slow_: 0.62 ± 0.02, *n* = 25; ALA: 18.36 ± 1.78 ms (τ_fast_), A_fast_: 0.42 ± 0.05, *n* = 13; 210.44 ± 21.28 (τ_slow_) A_slow_: 0.58 ± 0.05, *n* = 13; no significant FLU effect on percentages was observed] and **(b)** FR10 upon GABA+FLU application relative to control for CYS (for GABA: 0.97 ± 0.01, *n* = 9). **(G)** Typical normalized traces of current responses elicited by prolonged pulses revealing the differences in the time course of macroscopic desensitization (controls – black traces, responses in the presence of FLU – gray traces). **(H)** Statistics for the relative FLU effect on FR500 values (absolute values of FR500 in control conditions: LEU: 0.17 ± 0.02, *n* = 26; ALA: 0.18 ± 0.03, *n* = 16; CYS: 0.61 ± 0.05, *n* = 10). **(I)** Statistics for deactivation kinetics: **(a)** relative effect of FLU on the deactivation time constants [absolute values of deactivation parameters in control conditions: LEU: 24.67 ± 2.82 ms (τ_mean_); 3.32 ± 0.43 (τ_fast_); 40.96 ± 4.9 ms (τ_slow_), *n* = 12; ALA: 13.37 ± 1.5 ms (τ_mean_), *n* = 5; CYS: 16.99 ± 1.57 ms (τ_mean_), *n* = 8], **(b)** relative amplitude percentages for LEU deactivation kinetics (absolute values for GABA: A_fast_: 0.43 ± 0.03; A_slow_: 0.57 ± 0.03, *n =* 12), and **(c)** deactivation parameters after a long pulse [for GABA: LEU: 51.03 ± 10.07 ms (τ_mean_), *n* = 26; ALA: 21.61 ± 3.25 ms (τ_mean_), *n* = 14; CYS: 23.02 ± 3.7 ms (τ_mean_), *n* = 9]. For kinetic analysis of LEU and ALA, recordings were performed in the whole-cell configuration using the ultrafast perfusion system (theta glass) and to measure the amplitudes for CYS mutants, the Bio-Logic system was used. Asterisks show a statistically significant difference.

The impact of FLU on current responses mediated by saturating [GABA] was described by us for the α_1_β_2_γ_2_ receptors in our report (Mercik et al., 2007). Briefly, FLU slightly but significantly reduced the current amplitude; macroscopic desensitization was not affected and deactivation showed a slight and not significant prolongation. Similar effects were observed for cultured hippocampal neurons (Mozrzymas et al., 2007), which express primarily the α_1_β_2_γ_2_ receptors. Considering that α_1_F64 mutations strongly affect the receptor gating (particularly flipping/preactivation), we next investigated the impact of FLU on currents elicited by saturating [GABA] and mediated by these mutants. In contrast to WT receptors, current responses elicited by 100 mM GABA for CYS mutants were significantly potentiated by FLU in the pretreatment protocol (**Figures 3E, 4A,B**) and this effect was significantly larger when co-applying FLU with GABA (**Figure 3E**). In the case of the ALA mutant, the effect of FLU was weaker but potentiation in both pretreatment and co-application protocols was significant (**Figures 3E, 4B**). On the contrary, for the LEU mutant, we found that FLU slightly decreased the current amplitude in the pretreatment protocol but did not affect it when co-applied with GABA (**Figures 3E, 4B**). A similarity in the FLU effect on WT receptors and LEU mutants is consistent with our finding that this mutant showed the closest resemblance to the WT receptors among those considered in Szczot et al. (2014) and in the present study.

The observation that FLU exerted a qualitatively different effect on the current amplitudes for WT receptors and mutants (CYS and ALA) indicates a difference in receptor gating and we thus extended our analysis to the time course of these responses. Whereas FLU had no effect on the onset kinetics in the case of WT receptors (Mercik et al., 2007), a significant shortening of the current RT was observed for all considered α_1_F64 mutants (**Figures 4C,D**). Moreover, FLU affected the macroscopic desensitization primarily by accelerating its rapid component (**Figures 4E,Fa**, absolute values for desensitization and deactivation time course parameters in control conditions are disclosed in the Figure legend) for LEU and ALA mutants but not for CYS, for which it was assessed as the FR10 parameter that remained unaffected (**Figures 4E,Fb**). An increase in the FR500 parameter for LEU and ALA (but not CYS) mutants indicated the impact of FLU on the extent of desensitization (**Figures 4G,H**). The mean time constant for deactivation (τ_mean_) for currents elicited by short GABA pulses was not significantly affected by FLU in all mutants, but in the case of LEU, the fast component was accelerated together with a change in percentages for time constants (**Figures 4Ia,b**). In the case of deactivation measured after a prolonged application (500 ms) of saturating [GABA], no effect of FLU was found for all considered mutants (**Figure 4Ic**).

### Impact of Flurazepam on Currents Evoked by a Partial Agonist P4S

**FIGURE 5:**
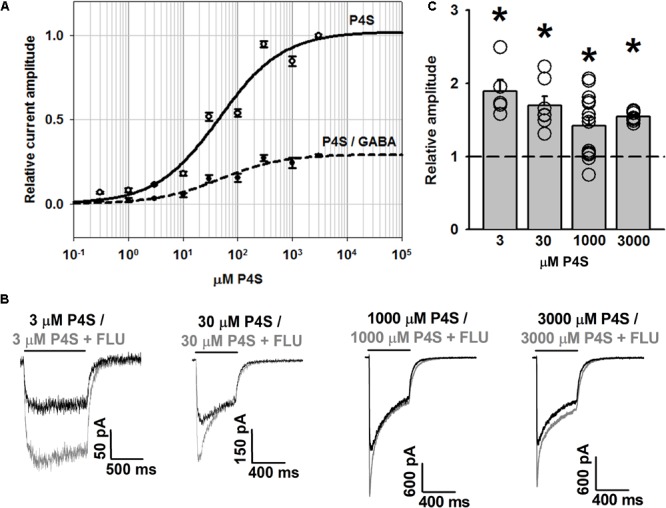
FLU potentiates WT GABA_A_R responses elicited by partial agonist P4S. **(A)** Dose–response relationships of GABA_A_Rs for P4S (solid line) and for P4S normalized to response to saturating [GABA] (dashed line). **(B)** Typical traces of responses to various concentrations of P4S with (gray traces) and without FLU (black traces). **(C)** Statistics for the relative effect of FLU on amplitudes of responses to P4S (in mM: 3, 30, 1,000, 3,000; *n* = 5, 7, 14, and 7, respectively). All recordings were performed in the whole-cell configuration using the ultrafast perfusion system (theta glass). Insets above current traces indicate agonist applications and asterisks statistical significance.

To further address the effect of FLU on GABA_A_R gating, we have analyzed its impact on responses elicited by a partial agonist P4S. As expected, the dose–response obtained for P4S showed saturation at current values considerably lower than for GABA but the values of EC50 did not show any major difference (EC50 = 46 μM for P4S, **Figure 5A**, compared to 40 μM for GABA determined by Brodzki et al., 2016). Notably, in contrast to our observations for GABA (Mercik et al., 2007), responses to saturating concentrations of P4S were clearly potentiated by FLU for the entire range of concentrations used (**Figures 5A–C**).

**FIGURE 6:**
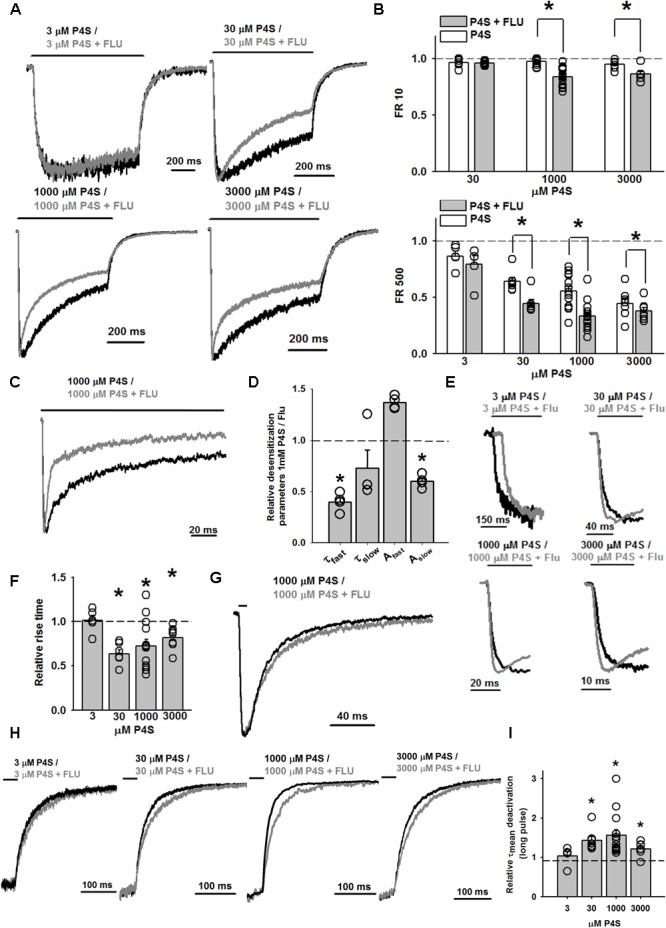
FLU markedly affects the kinetic profile of WT GABA_A_R-mediated currents evoked by partial agonist P4S. **(A)** Typical normalized traces of current responses to various concentrations of P4S, mediated by WT GABA_A_R in control conditions (black traces) and in the presence of FLU (gray traces). **(B)** Bar charts illustrating the statistics of the FLU effect on FR10 and FR500 parameters for responses evoked by P4S (in mM: 3, 30, 1,000, 3,000; *n* = 5, 7, 14, and 7, respectively). **(C)** Typical normalized traces recorded from outside-out patches containing WT GABA_A_Rs responding to 1 mM P4S alone (black trace) and in the presence of FLU (gray trace). **(D)** Relative macroscopic desensitization time course parameters for recordings from outside-out patches. Absolute values for control (1 mM P4S: τ_fast_: 4.14 ± 0.69 ms; τ_slow_: 197.07 ± 22.32 ms; A_fast_: 0.52 ± 0.02; A_slow_: 0.48 ± 0.02; *n* = 4) **(E)** Close-up, normalized traces of currents evoked by various concentrations of P4S alone (black traces) and in the presence of FLU (gray traces) with emphasis on the effect of FLU on the current onset. **(F)** Statistics for the relative FLU effect on the RT measured at various P4S concentrations. Absolute values of RT for control (3 μM: 71.98 ± 4.72 ms, *n* = 5; 30 μM: 20.35 ± 2.07 ms, *n* = 7; 1 mM: 6.79 ± 0.60 ms, *n* = 14; 3 mM: 5.89 ± 0.55 ms, *n* = 7). **(G)** Typical normalized traces of current responses to 1 mM P4S alone (black trace) and in the presence of FLU (gray trace). Note a slowdown of deactivation in the presence of FLU. **(H)** Close-up, normalized traces of currents evoked by various concentrations of P4S alone (black traces) and in the presence of FLU (gray traces) with emphasis on the effect of FLU on the current deactivation after prolonged agonist pulse. **(I)** Relative effect of FLU on deactivation time constants for responses to various concentrations of P4S, measured after prolonged agonist pulse. Absolute values of τ_mean_ for control (3 μM: 77.15 ± 5.32 ms, *n* = 5; 30 μM: 60.23 ± 5.39 ms, *n* = 7; 1 mM: 54.44 ± 6.23 ms, *n* = 14; 3 mM: 78.11 ± 7.59 ms, *n* = 7). All recordings were performed in the whole-cell configuration using the ultrafast perfusion system (theta glass). Insets above current traces indicate agonist applications and asterisks statistical significance.

Importantly, whereas FLU only slightly affected the time course of responses evoked by saturating [GABA] (Mercik et al., 2007), the kinetics of currents elicited by saturating [P4S] was clearly altered (**Figures 5B, 6A**). The most apparent effect of FLU on P4S-evoked responses was the enhancement of the macroscopic desensitization (**Figures 5B, 6A**). For responses to 30 μM P4S, FR10 was close to 1 (minimal desensitization) and was unaffected by FLU (data not shown). Likewise, at low [P4S] (3 μM), FR500 was not affected but at 30 μM and higher concentrations, a strong increase in the extent of macroscopic desensitization (FR500) was observed (**Figures 6A,B**). For responses evoked by saturating (1 mM) P4S, the exponential fit was possible and revealed that FLU robustly increased in the rate and extent of macroscopic desensitization (accelerated τ_fAst_ and decreased FR500, **Figures 6B–D**). Notably, the desensitization onset for P4S-evoked currents was much slower (even in the presence of FLU) than that for saturating [GABA] (Mercik et al., 2007).

As we have previously shown (Szczot et al., 2014), the onset kinetics for currents evoked by saturating [P4S] was much slower than that of the responses elicited by 10 mM GABA. However, whereas for saturating [GABA] FLU had no effect on RT (Mercik et al., 2007), we show here that this compound clearly accelerated it for responses evoked by P4S (30 μM–3 mM) including saturation (**Figures 6E,F**).

For short and long P4S pulses, deactivation kinetics was much faster than for GABA-evoked currents (for 1 mM P4S, short pulse: τ_mean_ = 20.59 ± 3.83 ms, *n* = 7, long pulse τ_mean_ = 54.44 ± 6.23 ms, *n* = 14; for GABA: τ_mean_ = 52.29 ± 24.77 ms, long pulse τ_mean_ = 232.69 ± 33.51 ms; *p* < 0.05 for τ_mean_ comparison for short and long pulses). When applying a short pulse of 1 mM P4S, FLU prolonged the deactivation kinetics (for FLU: τ_mean_ = 24.35 ± 2.84 ms, *n* = 7, *p* < 0.05, **Figure 6G**) and a qualitatively analogous effect was observed after a long (500 ms) pulse of P4S at various concentrations (**Figures 6H,I**).

Taking altogether, we reveal that the FLU effects on currents evoked by P4S are by far more pronounced than in the case of GABA. Moreover, at a qualitative level, the FLU effects on currents evoked by P4S show similarity to those observed for currents mediated by the α1F64 mutants.

### Model Simulations

**FIGURE 7:**
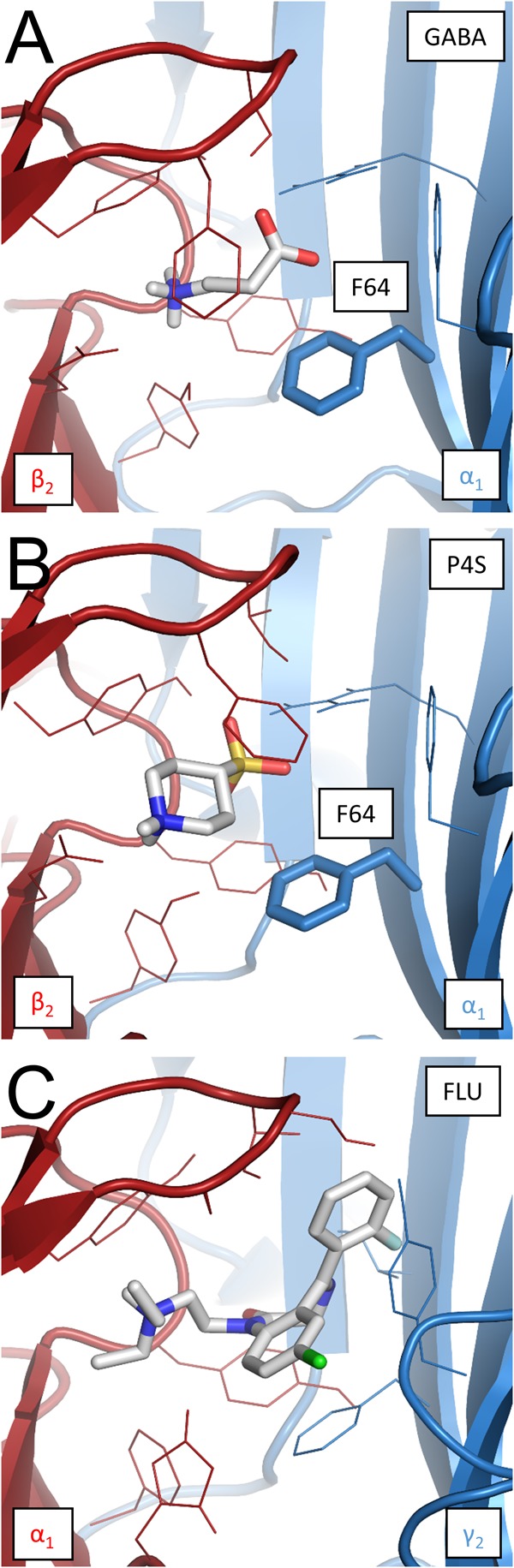
Binding modes of GABA, P4S, and FLU at the respective binding sites in the α_1_β_2_γ_2_ GABA_A_R homology model. **(A)** GABA at the β_2_/α_1_ interface binding site. Free energy of binding (ΔG) –5.2 kcal/mol, disassociation constant (K_d_) 1.54 × 10^-4^ M. α_1_F64 marked in bold stick representation. **(B)** P4S at the β_2_/α_1_ interface binding site; ΔG = –6.7 kcal/mol, K_d_ = 1.22 × 10^-5^ M. α_1_F64 marked as in the previous visualization. **(C)** FLU at the α_1_/γ_2_ interface binding site; ΔG = –8.6 kcal/mol, K_d_ = 4.93 × 10^-7^ M. Bigger size of FLU (387.88 g mol^-1^) compared to GABA (103.12 g mol^-1^) and P4S (165.21 g mol^-1^) results in a broader net of interactions with binding site residues and markedly lowers the disassociation constant.

To obtain further insight into the mechanisms whereby FLU modulates the spontaneous and ligand-induced GABA_A_R activity, model simulations were investigated. First, we asked whether very slow deactivation of spontaneous currents upon FLU application (**Figure 1**) is associated with ligand binding. To address this issue, the dissociation constant for this compound was assessed using homology modeling and docking studies, and the binding properties for GABA and P4S were also determined. The binding modes for considered compounds are presented in **Figures 7A–C** with estimated binding energies and disassociation constants, disclosed in the legend. Notably, the lowest disassociation constant value was for FLU, indicating a slow unbinding and deactivation kinetics.

**FIGURE 8:**
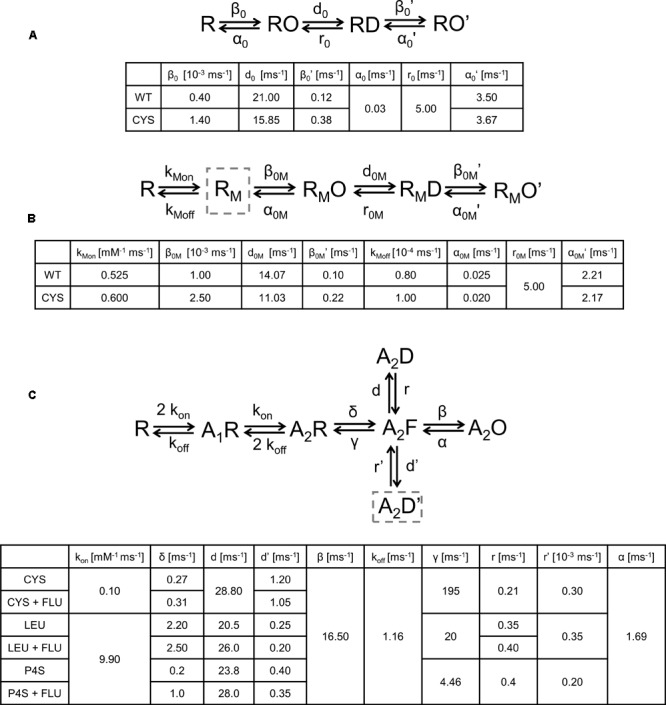
Kinetic scheme models of the GABA_A_R activation process and the values of transition rates affected by mutations or the ligand type. **(A)** Model describing spontaneous activity in the absence of modulator. Impact of CYS mutation was best modeled by decrease in α_0_ and d_0_ and increase in β_0_ and β_0_′. **(B)** Modulation of spontaneous activity by FLU. The impact of cysteine mutation modulated by FLU spontaneous activity was best reproduced by the following changes in the rate constants: β_0M_ and β_0M_′ increased and α_0M_ and α_0M_′ decreased, and desensitization rate d_0M_ decreased and a small change in binding rates was introduced. **(C)** Model for GABA-evoked activity. A_2_D is a fast desensitization state, highly pronounced in LEU mutant and P4S-evoked activation, whereas A_2_D′ is a slow desensitization state, most clearly visible as a slow macroscopic desensitization for each mutant and P4S-evoked currents for WT receptors. Exit transition rates from the slow desensitization state are particularly slow, resulting in experimentally observed particularly slow recovery kinetics (see **Figure 10**). Changes in rates for cysteine mutant after FLU application: δ increased and d′ decreased. For LEU mutant δ, d and r increased and d′ decreased. For P4S-evoked currents mediated by WT receptors changes in rates were the same as for LEU, except r which was not affected. Rates for WT receptor the same as in Szczot et al. (2014).

**FIGURE 9:**
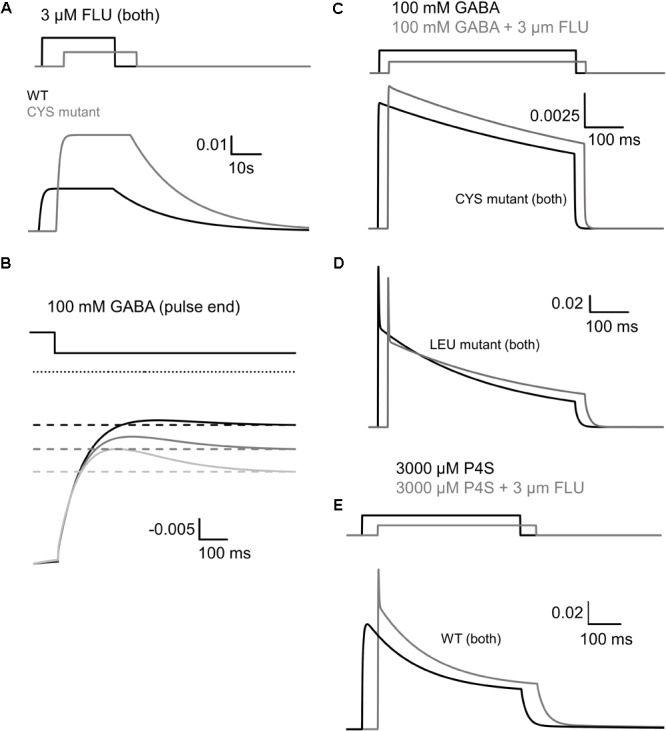
Simulated traces of spontaneous and ligand-evoked activity on the basis of kinetic schemes shown in **Figure 8**. **(A)** Enhancement of spontaneous activity of WT receptor (black) and CYS mutant (gray) by FLU. The relative effect of FLU was similar for both receptor types, but higher spontaneous activity of CYS mutant resulted in a larger increase in the absolute value of FLU-induced current. **(B)** Reproduction of the overshoot phenomenon and its dependence on the extent of spontaneous activity. Dotted line represents absolute zero probability and to make it easier to see the analogy to the experimentally observed overshoot, the graph is inverted. Time course of the open probability after agonist removal for WT receptor is shown with the black line. Upregulation of spontaneous activity (by increasing β_0_, mimicking FLU modulation or mutation, starting from WT level, gray traces) increases the amplitude of overshoot. **(C)** FLU Modulation of current responses to 100 mM GABA, mediated by CYS mutant. After modulator application, higher amplitude is visible but slow macroscopic desensitization remains unaffected. **(D)** Reproduction of FLU-induced modulation of GABA-evoked activity for the LEU mutant. Note a decrease in amplitude and fast desensitization acceleration (lower τ_fast_) but the slow desensitization component reduction results in increased FR500. **(E)** Reproduction of P4S-evoked activity and its modulation by FLU. Note the increased amplitude and more prominent desensitization, especially the appearance of the rapid component.

Spontaneous activity was modeled using the same kinetic scheme as proposed by Kisiel et al. (2018); **Figure 8A**. To model the FLU modulation of spontaneous activity, an additional state (RM, receptor with bound modulator) was introduced (**Figure 8B**) and the values of the rate constants were assessed as described in Section “Materials and Methods.” The effects of FLU modulation in both WT and CYS mutants were modeled by the decrease in α_m_, α_M_′, and d_m_, which are the rates determining the mean open times of the respective open states (**Figure 2E**). In addition to fully mimicking the FLU effects observed in macroscopic recordings, β_M_ was increased (**Figures 8A,B**, simulation in **Figure 9A**).

The fact that the overshoot was particularly prominent in the case of mutants (**Figure 3B**) suggests the involvement of spontaneous events. To further explore this issue, simulations were considered using the model proposed by (Szczot et al., 2014; **Figure 8C**) connected with the branch describing the spontaneous activity (**Figure 8A**). These simulations reproduced the dependence of the overshoot on the spontaneous activity (**Figure 9B**) and provided a mechanistic explanation for this phenomenon. The agonist pulse desensitizes the majority of receptors and thereby reduces the pool of spontaneously active channels and therefore, after agonist removal, the current exceeds the “baseline” (overshoot) and then slowly returns to the level prior to the agonist pulse, reflecting resensitization.

**FIGURE 10:**
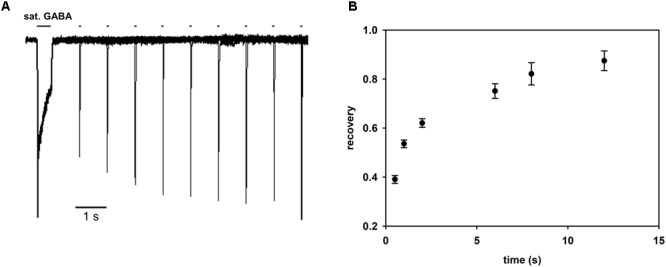
Recovery process following macroscopic desensitization induction by prolonged agonist pulse is very slow. **(A)** Superimposed exemplary traces of responses in the recovery protocol in which macroscopic desensitization is induced by a prolonged (500 ms) GABA pulse and, after a variable washout period, a test GABA pulse is applied (see section “Materials and Methods”). Exemplary recordings are shown for the LEU mutant. **(B)** Statistics of the extent of recovery against the wash interval duration calculated as (I_2peak_ – I_1end_)/(I_1peak_ – I_1end_), where I_1peak_ is the peak current value of the first pulse, I_1end_ the current value of the first response at the end of the agonist pulse, and I_2peak_ the peak current value of the second response. All recordings were performed in the whole-cell configuration using the ultrafast perfusion system (theta glass).

In several studies, the description of desensitization was limited to the fast component, in the range of a few milliseconds, claiming that this component is the most relevant to the time scale of synaptic events (e.g., Jones and Westbrook, 1995; Mozrzymas et al., 1999; Barberis et al., 2000). However, in the present study, the considered mutants show a prominent slow desensitization. We made an attempt to include this component in our modeling by adding additional desensitized transition, originating from the flipped state (**Figure 8C**). This transition allowed to fairly reproduce the slow component for the desensitization rate d′ considerably slower than d and a particularly slow resensitization rate r’ (**Figure 8C**). However, such values of d′ and r′ would predict a remarkably slow recovery. To provide experimental support for this prediction, we have performed recordings for LEU mutants in which a prolonged saturating GABA pulse, inducing macroscopic slow desensitization, is followed by a variable washout interval and a brief test pulse revealing the extent of recovery (**Figure 10**). As shown in **Figure 10**, these experiments confirm a very slow recovery predicted by the model.

Thus, to describe FLU effects on GABA-evoked currents mediated by mutants, the model with fast and slow desensitization states was considered (**Figure 8C**) that allowed to reproduce a very slow macroscopic desensitization for the CYS mutant due to mainly a slow desensitization transition, although a prominent overshoot for this mutant argues for a contribution from the fast component that is not manifested as a rapid fading due to extremely slow flipping (Szczot et al., 2014). In the case of the LEU mutant, a combination of slow and fast desensitization was necessary to reproduce our observations. Notably, the slow flipping rate in mutants (Szczot et al., 2014; Kisiel et al., 2018) made it possible to clearly observe the effect of upregulating these rate constants by FLU as a trend of changes toward the WT phenotype. The increased amplitude for the CYS mutant (**Figures 4A,B**) was modeled by increasing the flipping (δ) rate that also mimicked the acceleration of the current onset (**Figures 4C,D**, simulated response in **Figure 9C**). To maintain the correct value of FR500, d′ was decreased. The LEU mutant showed mixed effects of FLU action: fast desensitization was enhanced, whereas FR500 increased (**Figures 4F,H**), requiring an increase of d and r, and a decrease of d′, further confirming the need to include the two desensitization states. To reproduce a decreased amplitude and an accelerated RT for the LEU mutant (**Figures 4A–D**), the increase in d was not sufficient and, especially to correctly mimic the RT acceleration, an increase in δ (as for the CYS mutant) was needed (see simulated response in **Figure 9D**).

The activation of WT GABA_A_R by a partial agonist P4S is expected to occur with a reduced flipping rate with respect to GABA (Gielen et al., 2012; Szczot et al., 2014). Our major observation was that FLU tended to change the P4S-evoked currents toward the kinetic phenotype observed for GABA-evoked responses: most evidently, FLU accelerated the onset and macroscopic desensitization and increased the amplitude (**Figures 5, 6**). These effects could be fairly reproduced by increasing δ but to maintain the correct FR500 value, an increased d′, similar to the LEU mutant, was required (**Figure 9E**).

Note that in contrast to the α_1_F64 mutants, in the case of WT receptors, the FLU effect on currents evoked by saturating GABA was weak (Lavoie and Twyman, 1996; Mercik et al., 2007). To clarify this issue, we have run additional series of simulations for WT receptors activated by saturating GABA. We found that the flipping rate in these receptors is so fast that its further increase by FLU is not effective and the entry into the open states is counterbalanced by the entry into the rapidly desensitizing state, giving rise eventually to the decrease in current. Thus, a prominent impact of increased flipping on current kinetics in the case of mutants and P4S-evoked currents for WT receptors was possible because it was much slower than in the case of WT receptors.

## Discussion

In the present study, we present the first, to our knowledge, thorough analysis of a mechanism whereby a BDZ FLU affects the spontaneous activity of GABA_A_R. Our macroscopic and single-channel analyses indicate that FLU affects the opening/closing transitions, thereby reinforcing the notion that the BDZs affect the receptor gating. Moreover, upon prolonged exposure to FLU, the receptors undergo a slow macroscopic desensitization (**Figure 1Fb**) that demonstrates, for the first time, that the GABA_A_R may enter the desensitized state in the absence of the orthosteric agonist and this process may depend on BDZs. However, the percentage of unliganded desensitized GABA_A_Rs remains unknown. Our homology modeling indicated that a particularly slow deactivation kinetics after FLU removal (**Figure 1**) could result from a markedly slower unbinding rate for FLU compared to GABA or P4S. In addition, our data, obtained using the pretreatment and co-application protocols, provide a methodological hint that the spontaneous activity, especially in the presence of BDZs, may affect the readout of GABA-evoked responses.

Considering that the spontaneous activity represents a form of receptor gating, it might be surprising that the mutations located at the binding site (i.e., distantly from the gate) strongly affect it. However, the mutations at the binding site affecting spontaneous activity have been already described, e.g., the mutation at the β_2_E155 residue (Newell et al., 2004) or at the 102 residue in the ρ_1_ homomers (Torres and Weiss, 2002). It seems thus that the molecular structure of GABA_A_R macromolecule assures an efficient communication between distant localizations. Moreover, it is not surprising that the modulation of spontaneous activity by FLU for WT receptors and mutants was similar, as the kinetic features of this activity for these receptors are analogous (Kisiel et al., 2018).

Considering the impact of FLU on spontaneous GABA_A_R activity, two possible mechanisms could be considered: a direct activation by FLU or a modulatory effect on existing events. Our interpretation is leaning toward the second possibility for the following reasons. (i) There are two open time components in control conditions and in the presence of FLU also there were two components but with significantly different time constants (**Figure 2**). If FLU activated some extra openings, then we would expect some extra component(s) in the open time distributions (besides those found in the control recordings) that were actually not found. The fact that in the presence of FLU we see two components with different time constants provides an argument for a modulatory mechanism. (ii) The activation of extra events by FLU would be expected to increase the overall frequency of events while the open time components of control spontaneous activity should be preserved in the presence of this BDZ. As we already pointed out, this prediction is not observed, but in reference to the frequency issue we saw a trend toward an increase in the presence of FLU, although it did not reach significance due to the large data scatter. We believe that this trend to increase the frequency in the presence of FLU is likely to represent a lower percentage of undetected events due to a prolongation of the open times. Undoubtedly, there is a percentage of extremely short events that are at the border line of our resolution. Prolongation of openings by FLU increases the chances of detection of these short openings, giving rise to apparently a larger frequency of events with distinct open time distribution than in control conditions. On the other hand, we cannot exclude some modulatory effect of FLU on the opening rates (β) of spontaneous events.

Interestingly, the cross-talk between the spontaneous and GABA-evoked activity, manifested by the overshoot, is sensitive to the modulation of spontaneous openings by FLU. A similar overshoot phenomenon was reported previously by Wagner et al. (2005) for GABA_A_R containing the 𝜀 subunit. It seems interesting to extend the analysis of cross-talk between spontaneous and GABA-evoked activity to other receptor types, including those contributing to both tonic and phasic inhibition (like, e.g., GABA_A_Rs containing α5 subunit; Caraiscos et al., 2004; Zarnowska et al., 2009) or those mediating tonic currents. Our major conclusion regarding the mechanism of GABA_A_R modulation by FLU is that this compound enhances the flipping and desensitization rates pointing thus to a “mixed” effect. Our proposal that FLU upregulates the flipping rate is based on the experiments on α_1_F64 mutants and on the activation of the WT receptor by a partial agonist P4S. Indeed, in our model simulations for these two sets of experiments, most FLU effects could be mimicked by the increased flipping rate, which altered the current kinetics toward the WT receptor phenotype. On the other hand, the impact of FLU on currents mediated by α_1_F64 mutants and on P4S-evoked responses of WT receptors showed some differences (**Figures 4–6**). This could result from the different values of flipping rates in α_1_F64 mutants and WT receptors activated by P4S but also because, as shown in our recent report (Kisiel et al., 2018), the mutation at the α_1_F64 residue alters not only flipping but also other gating transitions. Moreover, although there is an emerging agreement that partial agonists differ from full ones primarily by the flipping transitions (Lape et al., 2008), it cannot be taken for granted that other rate constants are equal for P4S and GABA. Notably, besides a prominent effect of FLU on the flipping rate, our analysis indicates a clear effect on desensitization. It needs to be emphasized that a diversity of the effects of the BDZ on gating is manifested here additionally by the fact that FLU affects opening/closing for spontaneous events whereas flipping and desensitization are affected for GABA-evoked activation. It seems thus that FLU may exert a mixed effect on the receptor gating, requiring further studies using different agonists and different mutations.

Taking altogether, we demonstrate that FLU affects the gating properties of the α_1_β_2_γ_2_ GABA_A_R, but this modulation is characterized by a distinct mechanism in the case of spontaneous and GABA-evoked activity. Moreover, our analysis of spontaneous and agonist-evoked activity provides further evidence that the phenomenon of flipping requires an interaction with the agonist and the desensitization process may take place in the absence of the orthosteric ligand. This study extends our knowledge on the mechanism of action of BDZs in the context of the structure–function relationship of GABA_A_Rs, offering new perspectives in designing these drugs for clinical use.

## Data Availability

Datasets are available on request. Please contact corresponding authors.

## Author Contributions

MJ-Ś designed and performed the experiments, analyzed the data, and wrote the paper. KT performed the experiments, analyzed the data, and wrote the paper. MB participated in carrying out the experiments, analyzing the data, and writing the paper. MM performed homology modeling, ligand docking, kinetic simulations, and wrote the paper. MC performed the experiments and analyzed the data. JN and AA contributed to some experiments and performed a preliminary analysis. RS performed a part of single-channel experiments. JM conceived and supervised the project, designed the research, participated in data analysis and model simulations, wrote most parts of the paper and edited the final version of the manuscript, and procured financial support.

## Conflict of Interest Statement

The authors declare that the research was conducted in the absence of any commercial or financial relationships that could be construed as a potential conflict of interest. The handling Editor declared a past co-authorship with one of the authors, JM.

## Footnotes

1 http://www.onemol.org.uk/

## Abbreviations

ALA: α_1_F64A mutant of α_1_β_2_γ_2_ GABA_A_ receptor
BDZ: benzodiazepine
CYS: α_1_F64C mutant of α_1_β_2_γ_2_ GABA_A_ receptor
FLU: flurazepam, which is a benzodiazepine derivative
FR: fraction of the current that remained after the selected time after the peak
GABA_A_R: ionotropic GABAergic receptor, type A
LEU: α_1_F64L mutant of α_1_β_2_γ_2_ GABA_A_ receptor
P4S: piperidine-4-sulfonic acid
PTX: picrotoxin, open GABA_A_ receptor channel blocker
RT: rise time of currents mediated by GABA_A_ receptors upon activation
WT: wild-type of α_1_β_2_γ_2_ GABA_A_ receptor
